# Lenvatininb as Treatment for Naïve Patients with Aggressive Thyroid Cancer Bone Metastases and Bad Performance Status

**DOI:** 10.1155/2020/8679149

**Published:** 2020-06-04

**Authors:** Sonia Garcia-Rodriguez, Guillermo Martinez Pinillos, Manuel Chaves-Conde

**Affiliations:** ^1^Medical Oncology Department, Hospital Nuestra Señora de Valme, Seville, Spain; ^2^Endocrinology and Metabolism Department, Hospital Nuestra Señora de Valme, Seville, Spain

## Abstract

Lenvatinib is an oral multityrosine kinase inhibitor (TKI) with proven effectiveness in the treatment of radioactive iodine- (RAI-) refractory and/or unresectable differentiated thyroid carcinoma (DTC). The present study reports the case of a 41-year-old male who underwent hemithyroidectomy in June 2015 due to a thyroid nodule with fine-needle aspiration follicular neoplasm cytology and no evidence of malignancy in the histopathological exam. Three years later, acute disabling clinical symptoms emerged, mainly high skeletal pain conditioned with an important performance status decrease. PET/CT scan displayed several metastatic bone lesions in this context, located in the vertebral bodies, sternum, ribs, iliac crest, right acetabulum, and both necks of the femur. Histological study and immunohistochemistry confirmed DTC metastases, as they were strongly positive for thyroglobulin and TTF-1. At this point, the patient was unfit for conventional management that would have included completion of surgery and RAI treatment as first options. Thus, it was decided to start systemic treatment with TKI, Lenvatinib. Within the first week of treatment, the patient was almost asymptomatic and his performance status moved from 3 to 0. This allowed the patient to undergo resection of the thyroid gland remnant plus RAI treatment. Unfortunately, RAI refractory illness was confirmed so Lenvatinib treatment should be continued in this case until the evidence of no further clinical benefit. Despite drug adverse events, the patient continues with treatment one year later, remaining asymptomatic and with normal functional capacity.

## 1. Introduction

Hematogenous metastasis from differentiated thyroid carcinoma (DTC) typically appears in the lung (49%) and bone (25%) [1]. The spine is the commonest site of bone metastasis, followed by the pelvis, skull, long bones, and sternum [2]. However, compared with lung involvement, patients with bone metastasis have generally a worse prognosis. While the 10-year survival rates up to 95% in localized DTC, the rate for bone metastatic scenario drops down further to 13-21% before tyrosine kinase inhibitor (TKI) era [3]. Management of metastatic disease generally include surgery, radioactive iodine (RAI) therapy, external beam radiation, and subsequently oral systemic treatments with tyrosine kinase inhibitors (TKIs) [4]. Among these, Lenvatinib is an oral multi-TKI of vascular endothelial growth factor (VEGF) receptors 1-3, fibroblast growth factor receptor alpha (PDGFR-*α*), ret proto-oncogene (RET), and KIT proto-oncogene. Due to this, mechanism inhibits tumour angiogenesis with proven effectiveness in the treatment of DTC with RAI-refractory and/or unresectable progressive disease [5]. In the phase III SELECT study, Lenvatinib, as compared with placebo, was associated with significant improvements in progression-free survival and the response rate in patients with progressive thyroid cancer refractory to iodine-131 [6].

## 2. Case Report

The present study reports the case of a 41-year-old male. He had no history of tobacco or alcohol abuse, neither chronic medical condition. He was diagnosed a nonfunctioning 7 cm left-sided thyroid nodule. Fine-needle aspiration cytology reported the lesion as suspicious for a follicular neoplasm (Bethesda category IV) and subsequently underwent hemithyroidectomy, with left recurrent laryngeal nerve palsy as postoperative complication. Histopathological exam reported the presence of a follicular adenoma without any evidence of malignancy. In the follow-up visits, no evidence of illness was found within the next 36 months.

In November 2018, disabling acute symptoms emerged, such as fatigue, anorexia, and skeletal pain which required a high dose of analgesic drugs. At that moment, ^18^F-FDG positron emission tomography (PET)/CT scan displayed several bone lesions (vertebral bodies including C6-C7, sternum, ribs, iliac crest, right acetabulum, and both necks of the femur), with maximum standardized uptake value (SUV) of 13.4. Echography percutaneous biopsy was used to obtain a sample from rib lesion which histological examination by immunohistochemistry revealed evidence of DTC metastases as they were strongly positive for thyroglobulin and thyroid transcription factor 1 (TTF1).

After establishing diagnosis, the case was reviewed in a multidisciplinary tumour board. As the patient presented poor performance status at the moment of diagnose (ECOG 3), he was not candidate for receiving local treatments, such as completion of surgery. Also, RAI therapy at this moment was not suitable for several reasons. Firstly, bone metastasis presented ^18^F-FDG uptake with high SUV, for that reason less likely to concentrate radioiodine and high probability of being RAI refractory. Secondly, it was considered to be a high risk of vertebral fracture and spinal cord compression at the level of C6-C7. After a careful analysis of these clinical features, it was decided to start systemic treatment with TKI Levantinib. Oral treatment was administered continually in a 24 mg once daily-dose, for 4-week cycles.

**Figure 1:**
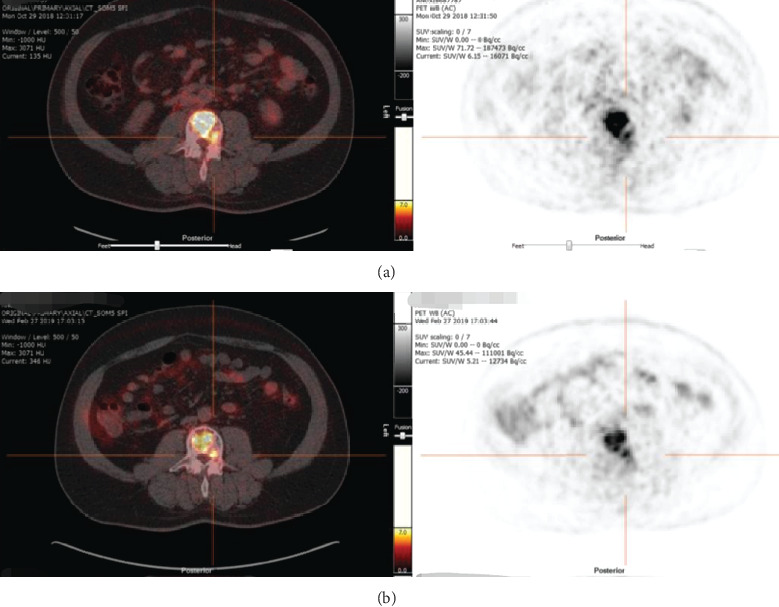
^18^F-FDG PET/CT scan showing the difference in SUV maximum for lesion located in C6 at the moment of diagnosis (a) vs SUV maximum for same location after 3-cycle of Lenvatinib oral treatment (b). The decrease was quantified in 39.5%.

One week after treatment initiation, the patient experienced remarkable improvement in his clinical condition, including high reduction of skeletal pain that allowed a decrease in the analgesic treatment as well as a significant improvement of patient's performance status, moving from ECOG 3 to 0. After three cycles of treatment, a new ^18^F-FDG PET/CT scan was performed for restaging. It was observed a reduction in the size of lesions, mainly in the soft tissue component and a decrease of maximum SUV from 13.4 to 8.1 (Figure 1), which represents a 39.5% of decrease. Serum thyroglobulin levels also decreased from 49105 ng/ml to 2500 ng/ml in this period.

In view of this clinical and radiological response, conventional treatment was suitable in this case, including completion of thyroid surgery plus RAI treatment. The patient underwent resection of the thyroid gland remnant, with no post operative complications and no evidence of malignancy on the histopathological exam. After surgery, RAI treatment was performed. In the cancer uptake scan, a low dose of radioiodine detected disease only in tumour bed as well as isolated vertebral metastasis. Afterwards, when a higher dose for ablation was administered, the scan showed also multiple foci of metastasis in the axial and peripheral skeleton. This absence of RAI uptake in some but not in all lesions confirmed the presence of dedifferentiated thyroid cancer population (with low ability to uptake and concentrate radioiodine) and therefore RAI-refractory disease.

Regarding adverse events (AEs), the patient experienced hypertension within the first days of treatment, later followed by diarrhoea, both grade III according to the Common Terminology Criteria for Adverse events (CTCAE) scale. He also experienced transient low-grade stomatitis that was spontaneously resolved. With respect to hypertension, early blood pressure control was started as a method to maintain TKI dose regimen. Patient was clearly instructed to report side effects when they are still low grade in order to avoid TKI interruption. It could be optimally controlled with calcium antagonist and ACE inhibitors, and no dose reduction was needed for this reason. Due to diarrhoea, dose reduction as well as one-day dose interruption once or twice per cycle was needed, in addition to antidiarrhoeal medication such as loperamide. Late adverse events observed after one year of treatment were neutropenia grade II and moderate to severe palmar-plantar erythrodysaesthesia that lead us to a dose reduction up to 10 mg once daily-dose for 3-week cycles followed by 1 week off-treatment.

## 3. Discussion and Conclusion

At the moment of diagnosis, the patient presented multiple bone metastasis as well as high burden of symptoms (fatigue, anorexia, and disabling skeletal pain) that conditioned a low level of performance status (ECOG 3). This clinical situation prevented a conventional management that would initially include completion of thyroid surgery and RAI therapy as first options [7]. Thus, it was decided to start systemic therapy with Lenvatinib, carefully monitoring and early managing emerging adverse events.

After only 1 week of treatment, the patient experienced a remarkable improvement in his clinical condition, with ECOG moving down from 3 to 0. This improvement was stable after 8 months of Lenvatinib treatment and made completion of thyroid surgery plus RAI treatment suitable for this patient. RAI therapy remains the main treatment modality for DTC distant metastasis, although bone lesions are less likely to concentrate radioiodine compared with lung or other metastasis locations. Efficacy of RAI in bone metastasis context is estimated at 55% [8]. In our case, RAI-refractory illness was confirmed. It is impossible to elucidate if emergent use of Lenvatinib (before RAI treatment) has influenced cell population ability to uptake and concentrate radioiodine.

Regarding safety profile, AEs showed correspond with those found in the phase III SELECT study [6] and also in subsequent real-life studies. It is described a risk of rapid progression (within a few days) after discontinuation of Levatinib [9], so a big effort was made to avoid dose reduction or treatment interruption due to AEs. This is probably due to TKI mechanism of action, as they are cytostatic and not cytotoxic drugs, that exerts a break to the cell growth that can restart immediately when the break is removed [10].

In conclusion, Levantinib emergent use has proven to be an effective treatment option for recovering performance status as well as for radiological illness control also enabling to perform another therapeutic approach, such as surgery and RAI treatment. Due to RAI refractory scenario, RAI therapy is not expected to be fully effective for disease control in this case and Levantinib treatment should be continued until the evidence of no further clinical benefit.
